# Ridge-furrow with plastic film and straw mulch increases water availability and wheat production on the Loess Plateau

**DOI:** 10.1038/s41598-018-24864-4

**Published:** 2018-04-25

**Authors:** Gaoyuan Liu, Yuhuan Zuo, Qi Zhang, Lili Yang, Erlong Zhao, Lianyou Liang, Yan’ an Tong

**Affiliations:** 1 College of Natural Resources and Environment, Northwest A&F University, Yangling, 712100 Shaanxi China; 2 Fuping Comprehensive Experiment Station of Northwest A&F University, Weinan, 714000 Shaanxi China

## Abstract

Mulching is critical for increasing water availability and hence winter wheat production in dryland farming systems. A two-year study was conducted to assess the effects of mulches on soil water storage (SWS), temperature, water use efficiency (WUE) and yields of winter wheat on the Loess Plateau. Four treatments were examined: conventional flat planting (CK), straw mulch (FPS), transparent plastic film mulch (FPP) and ridge-furrow with plastic film-mulched ridge and straw-mulched furrow (RFPS). Compared with CK, RFPS greatly increased SWS from 0–60 cm, FPP increased SWS from 0–40 cm, and FPS slightly increased SWS from 0–60 cm; however, FPP significantly (*P* < 0.05) decreased SWS from 61–100 cm. RFPS and FPP increased soil temperatures in cold seasons relative to CK, especially in RFPS (2.0–2.3 °C). Meanwhile, the rate of soil temperature increase was greater in RFPS and FPP than in CK but was lower in FPS. Mean yields were significantly increased in RFPS (56.78%), FPP (44.72%) and FPS (9.57%), and WUE was significantly increased in RFPS (44.04%) and in FPP (37.50%) compared with CK (*P* < 0.05). We conclude that ridge-furrow planting with plastic film-mulched ridge and straw-mulched furrow has a good potential for raising winter wheat production on the Loess Plateau.

## Introduction

Drylands cover approximately 45% of Earth’s land surface^1^, and 57% of the world’s potentially productive area is located in drylands, which are home to 41% of the world’s population-approximately 2 billion people^2^. Drylands will continue to produce most of the world’s food grains for expanding populations in the years ahead^3^; however, crop yields on drylands are tremendously low relative to those in humid and sub-humid regions^4^. In some countries of Africa and the Near East, food grain production per capita has declined significantly during the past decade^5,6^. Water scarcity is the main limitation to production in dryland farming systems^7^, and therefore, the importance of agronomic practices to increase water use efficiency (WUE) and to ensure the sustainability of dryland farming systems^8^, especially in arid and semiarid regions^9–12^, such as southeast Asia, Australia, and southern Africa, are important. In China, dryland farming is practised on approximately one-third of all arable land. Approximately 40% of this dry arable land is situated on the Loess Plateau^13^, which covers an area of 624,000 ha and is mostly important for cereal production^14,15^. In this region, mean annual precipitation ranges from 150–300 mm in the north to 500–700 mm in the south, with a 35% coefficient of variation^16^. In many locations in the plateau region, precipitation has declined over the last five decades at a rate of 1 to 2 mm yr^−1 ^^17^. Most crops planted in this region depend solely upon rainfall^18^, and are thus commonly drought stressed with low soil water storage (SWS). To produce sufficient food for a growing population, finding ways to improve crop WUE and thus sustain agricultural production is crucial.

Winter wheat (*Triticum aestivum* L.) is one of the most common crops on the Loess Plateau, occupying approximately 44% of the cultivated area, and plays a critical role in ensuring food security^19^. Being entirely dependent on precipitation, the winter wheat yield varies greatly from year to year and with precipitation^16^. Winter wheat is sown in late September and harvested in early June the following year. In this region, more than 60% of annual rainfall occurs during the July-September period, i.e., after harvest and before sowing. Clearly, the winter wheat growing season aligns poorly with the rainfall pattern^20,21^. Over the last 20 years, winter wheat yields have been increased by fertilizer application. However, this has resulted in a progressive depletions of SWS^22^. Some studies have reported that the drylands require higher and more stable water availabilities to produce higher winter wheat yields^16,23,24^. Therefore, the research aimed at increasing SWS and WUE has the potential to be highly beneficial for dryland farming systems. Likewise, the measures are needed to increase the harvesting of rainfall and its storage in root zone^25^.

Technologies for improving SWS and WUE are critical to sustainable crop production and food security in drylands^12,26^. A number of management practices have been demonstrated not only to decrease evaporation and improve water availability but also to increase crop productivity in semiarid regions. These practises include rainwater harvesting and mulching (including with plastic film, straw and gravel-sand^14,27–29^). Plastic film and straw mulches are the most common water conservation measures in arable dryland farming systems. However, the lower soil temperature caused by a straw mulch freezes the seedlings and roots of winter wheat in the cold winter months. This impacts both germination and tiller growth and hence tends to reduce crop production^30,31^. Moreover, although straw mulch reduces evaporation and improves SWS, these beneficial effects are both quite limited and very short lived^32^. These negative effects have limited the hopes for gains in crop productivity^23,33^. Many studies have shown that WUE and crop production using plastic film mulch are significantly increased as a result of improved SWS and increased soil temperatures^28,34^. Furthermore, ridge-furrow with a plastic-film mulch system has recently been used to increase rainfall harvesting and percolation. However, this practice carries with it a very high risk of surface crusting if heavy rainfall occurs before crop emergence^32^. Moreover, this method is not effective in decreasing soil moisture loss through surface evaporation in the furrow where crop is sown -especially early in the production cycle. Studies on the Loess Plateau have shown that this surface evaporation leads to significant soil water depletion when wheat shoots are small^13,14,16^.

The combination of the ridge covered by plastic film and the furrow covered by crop straw (RFPS) has been shown effective in improving SWS, in reducing soil evaporation and in increasing crop transpiration, the latter being associated with sustainable increases in crop productivity^34^. The effectiveness of such a management system has been well evaluated, and this system is strongly recommended for maize production^15,35^. However, little information is available for dryland winter wheat, and available results for the Loess Plateau, China, are not consistent^14,30,34^. Therefore, it is important to determine the effectiveness of this mulch practice and the productivity gains associated with its use with dryland winter wheat.

The objectives of this study were as follows: (1) to examine the practicability and reliability of RFPS relative to other mulching cultivations for dryland winter wheat on the Loess Plateau; (2) to differentiate between the influences of mulches on soil water availability and on soil temperature; (3) to assess the effects of different mulching cultivations on winter wheat yield, total water consumption (ET) and WUE; and (4) to analyse the economic benefits of different mulching cultivations for wheat farmers. The results of this study will provide a theoretical basis for the development of improved management practices for winter wheat production and water utilization in dryland farming systems.

## Results

### Soil moisture dynamics

**Figure 1:**
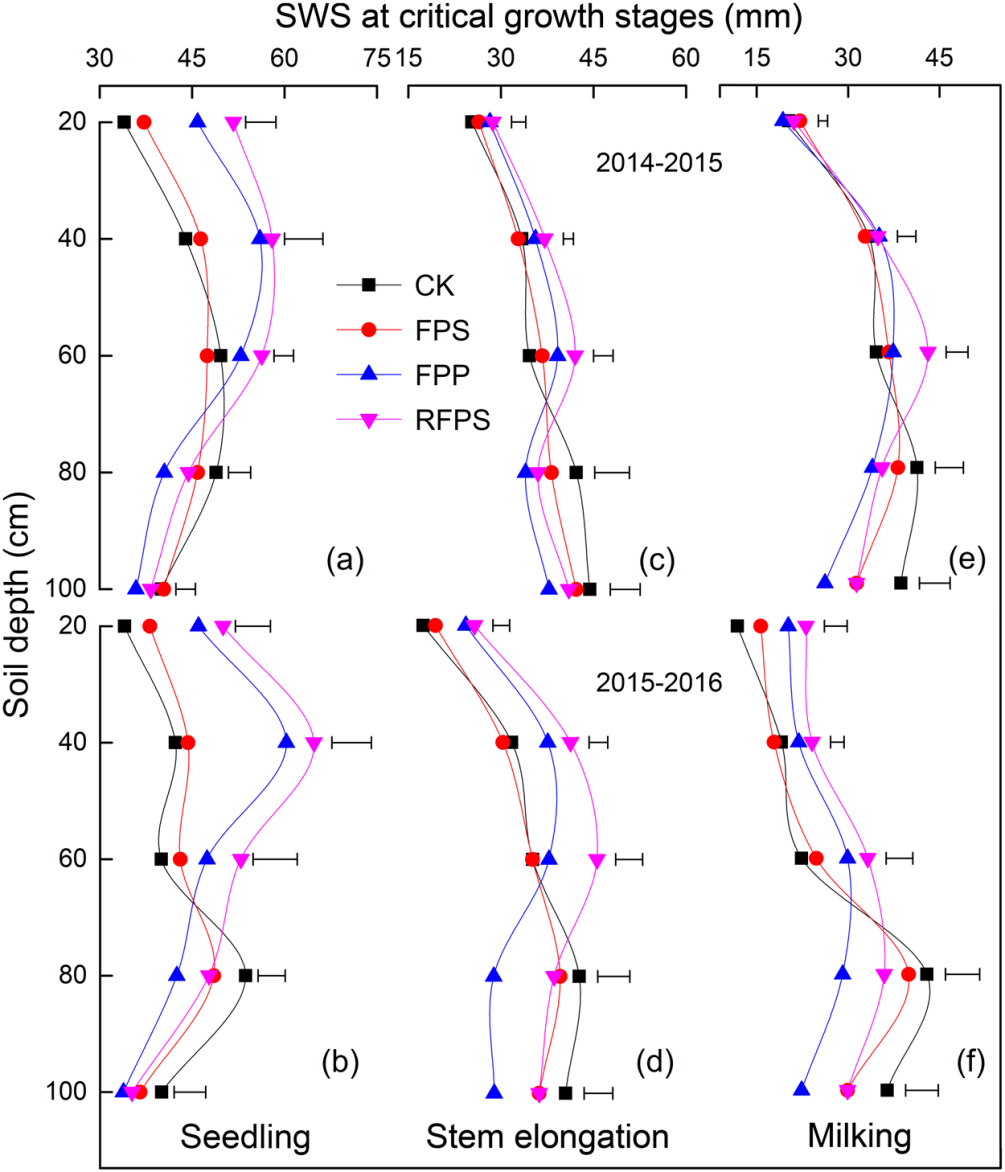
Effects of different mulching cultivations on soil water storage at a 20 cm depth interval within 100 cm at critical growth stages of winter wheat. (**a**), (**b**) and (**c**) represent soil water storage at the seedling (BBCH 17), stem elongation (BBCH 33) and milking (BBCH 73) stages in 2014–2015, and (**d**), (**e**) and (**f**) represent those stages in 2015–2016, respectively. CK, conventional flat planting; FPS, straw mulch; FPP, transparent plastic film mulch; RFPS, ridge-furrow with plastic film-mulched ridge and straw-mulched furrow. Vertical bars stand for LSD_0.05_ between different treatments (n = 3).

**Figure 2:**
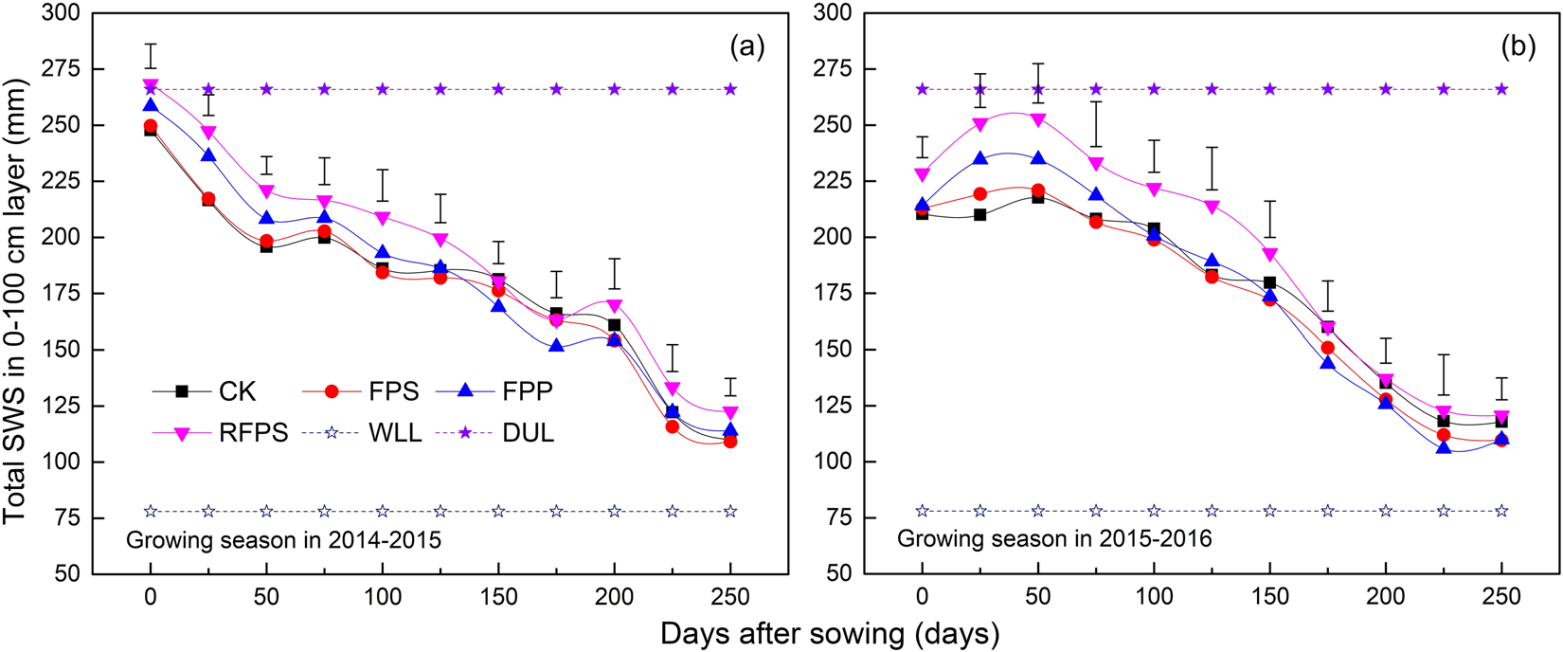
Effects of different mulching cultivations on total soil water storage from 0–100 cm over both growing seasons of winter wheat. (**a**) and (**b**) represent soil water storage in 2014–2015 and 2015–2016 growing seasons, respectively. CK, conventional flat planting; FPS, straw mulch; FPP, transparent plastic film mulch; RFPS, ridge-furrow with plastic film-mulched ridge and straw-mulched furrow. Vertical bars stand for LSD_0.05_ between different treatments (n = 3).

Soil water storage was clearly affected by soil depths, growth seasons and mulch treatments in both growing seasons (Figs 1 and 2, Supplementary Fig. S1). At the seedling stage (BBCH 17), the average annual SWS in RFPS and FPP was significantly (*P* < 0.05) higher than that in FPS (35.25% and 25.42%) and CK (45.57% and 35.00%) from 0–40 cm; that in RFPS was significantly (*P* < 0.05) increased by 20.67% and 21.86% compared with FPS and CK from 41–60 cm (Fig. 1a,b). At the stem elongation stage (BBCH 33), from 0–40 cm, the average annual SWS in RFPS and FPP was 21.01% and 14.76% higher than that in FPS and was 22.18% and 15.87% higher than that in CK, respectively; that in RFPS was significantly (*P* < 0.05) increased by 21.26% and 24.68% compared with FPS and CK from 41–60 cm (Fig. 1c,d). At the milking stage (BBCH 73), the average annual SWS in RFPS was significantly (*P* < 0.05) increased by 26.64% compared with that in CK from 0–60 cm, but there were no significant differences (*P* > 0.05) among FPP, FPS and CK (Fig. 1e,f). In addition, from 61–100 cm, the FPP significantly (*P* < 0.05) decreased the average annual SWS compared with CK, i.e., by 16.40% at the seedling stage, by 23.07% at the stem elongation stage, and by 29.85% at the milking stage, but no significant difference (*P* > 0.05) was found among RFPS, FPS and CK (Fig. 1).

Overall, the SWS from 0–100 cm under all treatments declined steadily from sowing to harvest (Fig. 2). In both growing seasons, the RFPS, FPP and FPS consistently retained more water than CK from 0 to 125 days after sowing (DAS), with the effectiveness being in the order of RFPS > FPP > FPS, but there were no obvious differences among all treatments from 150 to 250 DAS.

### Soil temperature

**Figure 3:**
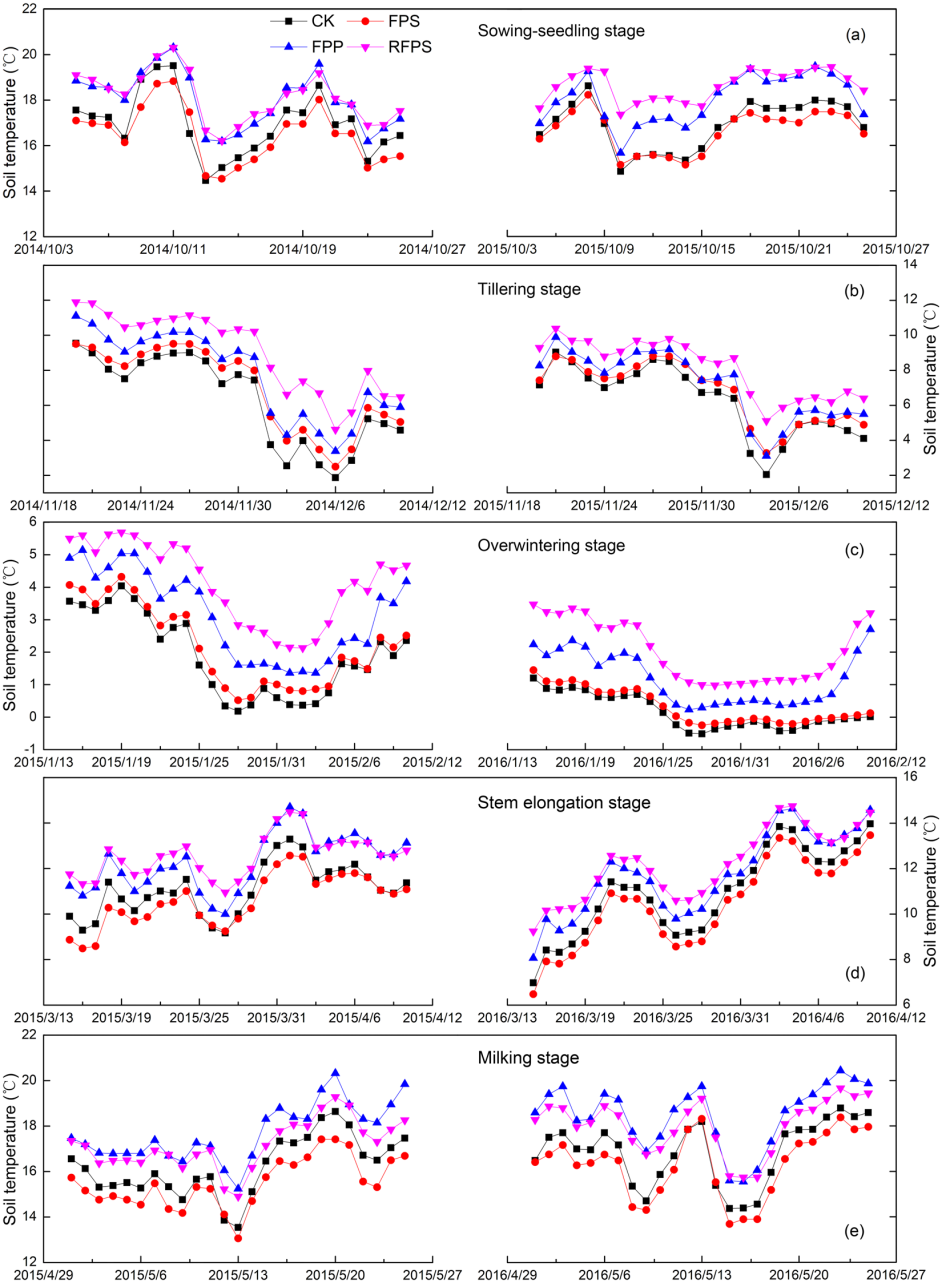
Effects of different mulching cultivations on daily mean soil temperature at 10 cm soil depth over both growing seasons. (**a**), (**b**), (**c**), (**d**) and (**e**) represent daily mean soil temperature at the sowing-seedling (BBCH 00–19), tillering (BBCH 21–28), overwintering (BBCH 29–30), stem elongation (BBCH 31–39) and milking (BBCH 73–77) stages, respectively. CK, conventional flat planting; FPS, straw mulch; FPP, transparent plastic film mulch; RFPS, ridge-furrow with plastic film-mulched ridge and straw-mulched furrow.

The response of mean daily soil temperature to the mulch treatments was different in both growing seasons (Fig. 3, Supplementary Table S1). In comparison with CK, the RFPS and FPP increased the mean daily soil temperature by 1.5 and 1.1 °C from sowing to the seedling stage (BBCH 00–19), by 2.4 and 1.2 °C at the tillering stage (BBCH 21–28), by 2.2 and 1.1 °C at the overwintering stage (BBCH 29–30), by 1.5 and 1.0 °C at the stem elongation stage (BBCH 31–39), and by 1.1 and 1.6 °C at the milking stage (BBCH 73–77). The mean daily soil temperature under FPS was increased by 0.6 and 0.3 °C at the tillering and overwintering stages, whereas it was decreased by 0.6, 0.5 and 0.7 °C from sowing to the seedling stage and at the stem elongation and milking stages, respectively, relative to CK (Fig. 3, Supplementary Table S1). Overall, mean daily soil temperatures under RFPS and FPP were constantly higher than under CK over both growing seasons, especially in cold periods (tillering and overwintering stages) (Fig. 3, Supplementary Table S1). Furthermore, both RFPS and FPP accelerated the increases in soil temperature during warm periods, especially from sowing to the seedling stage and at the stem elongation stage, with RFPS peaking at the stem elongation stage and FPP peaking at the milking stage. The daily soil temperatures under FPS were 0.2–0.7 °C warmer than those under CK in cold periods and increased more slowly during the stem elongation and milking stages, remaining consistently lower than under CK.

### Grain yield and yield components

The overall yield of winter wheat was strongly affected by mulch treatments in both seasons (Table 1). In comparison with CK, under RFPS, FPP and FPS, overall yields were significantly (*P* < 0.05) increased by 56.04%, 43.58% and 8.08% in 2015 and by 57.55%, 45.91% and 11.06% in 2016, respectively, and there was a significant difference (*P* < 0.05) between different mulch treatments.

**Table 1:** Differences in yields and yield components of winter wheat in various mulching cultivations.

Years	Treatments	Ears m^−2^	Grain numbers ear^−1^	Grain numbers m^−2^	Grain weight (mg)	Grain yield (kg ha^−1^)
2014–2015	CK	340.93 ± 7.73 d	27.81 ± 1.54 b	9,481.26 ± 211.32 d	40.61 ± 0.56 c	3846.96 ± 182.78 d
	FPS	359.10 ± 11.48 c	28.10 ± 1.27 b	10,090.71 ± 332.06 c	41.17 ± 0.62 c	4157.70 ± 175.24 c
	FPP	422.31 ± 10.22 b	30.75 ± 1.46 a	12,986.03 ± 494.14 b	42.52 ± 0.37 b	5523.44 ± 214.91 b
	RFPS	440.47 ± 9.71 a	31.57 ± 1.78 a	13,902.48 ± 402.75 a	43.24 ± 0.32 a	6002.76 ± 223.67 a
2015–2016	CK	349.28 ± 6.13 d	26.15 ± 1.41 b	9,133.67 ± 468.79 d	40.28 ± 0.35 c	3678.50 ± 152.63 d
	FPS	364.53 ± 8.68 c	27.40 ± 1.04 b	9,988.12 ± 293.22 c	40.87 ± 0.28 c	4085.18 ± 187.10 c
	FPP	438.52 ± 7.30 b	29.54 ± 1.08 a	12,953.88 ± 397.26 b	41.43 ± 0.22 b	5367.24 ± 203.76 b
	RFPS	458.51 ± 8.92 a	29.98 ± 1.63 a	13,746.13 ± 354.07 a	42.24 ± 0.31 a	5795.39 ± 192.32 a

CK, conventional flat planting; FPS, straw mulch; FPP, transparent plastic film mulch; RFPS, ridge-furrow with plastic film-mulched ridge and straw-mulched furrow. Values are given as the means ± standard errors of means. Values followed by different letters in each column for each year represent significant differences (*P* < 0.05) between treatments based on LSD tests (n = 3).

#### Ears m^−2^

The ears m^−2^ under RFPS, FPP and FPS were significantly increased by 29.17%, 23.87% and 5.33% in 2015 and by 32.10%, 26.23% and 4.48% in 2016 compared with CK, respectively, and there was a significant difference among RFPS, FPP and FPS (*P* < 0.05).

#### Grain numbers ear^−1^

Compared with CK, the grain numbers ear^−1^ under RFPS and FPP were significantly (*P* < 0.05) improved by 13.52% and 10.57% in 2015 and by 14.65% and 12.96% in 2016; meanwhile, the grain numbers ear^−1^ under RFPS and FPP were significantly (*P* < 0.05) higher than those under NPS. However, no significant difference (*P* > 0.05) was observed between FPS and CK.

#### Grain weight

The grain weight under RFPS and FPP were significantly (*P* < 0.05) increased by 6.48% and 4.70% in 2015 and by 4.87% and 2.86% in 2016 compared with CK; but, there was no significant difference (*P* > 0.05) between FPS and CK. The grain weight of mulch treatments were ranked in the order, RFPS > FPP > FPS (*P* < 0.05).

### Total water consumption (ET) and water use efficiency (WUE)

The analyses of ET and WUE under different mulching cultivations are shown in Table 2. Compared with CK, the ET in RFPS, FPP and FPS significantly (*P* < 0.05) increased by 9.31%, 5.69% and 3.16% in 2015, and by 8.40%, 4.85% and 3.58% in 2016, respectively. The ET under RFPS was significantly (*P* < 0.05) improved by 3.43% and 5.96% in 2015 and by 3.39% and 4.65% in 2016 compared with those under FPP and FPS, respectively. In comparison with CK, the WUE in RFPS and FPP was significantly (*P* < 0.05) increased by 42.75% and 35.85% in 2015 and by 45.33% and 39.15% in 2016, respectively; however, no significant difference (*P* > 0.05) was observed between FPS and CK. Among all mulch treatments, the WUE in RFPS was significantly increased by 5.08% and 36.31% in 2015 and by 4.40% and 35.52% in 2016 compared with those in FPP and FPS, respectively, and that in FPP was significantly higher than in FPS (29.73%) (*P* < 0.05).

**Table 2:** Comparisons of total water consumption (ET) and water use efficiency (WUE) under different mulching cultivations.

Years	Treatments	Rainfall (mm)	ΔSWS (mm)	ET (mm)	WUE (kg ha^−1^ mm^−1^)
2014–2015	CK	194.90	117.71 ± 3.43 d	312.61 ± 3.43 d	12.31 ± 0.49 c
	FPS		127.59 ± 3.82 c	322.49 ± 3.82 c	12.89 ± 0.61 c
	FPP		135.48 ± 4.69 b	330.38 ± 4.69 b	16.72 ± 0.37 b
	RFPS		146.80 ± 4.21 a	341.70 ± 4.21 a	17.57 ± 0.52 a
2015–2016	CK	208.10	92.51 ± 2.82 d	300.61 ± 2.82 d	12.24 ± 0.35 c
	FPS		103.28 ± 2.09 c	311.38 ± 2.09 c	13.12 ± 0.29 c
	FPP		107.10 ± 2.17 b	315.20 ± 2.17 b	17.03 ± 0.32 b
	RFPS		117.77 ± 3.61 a	325.87 ± 3.61 a	17.78 ± 0.40 a

CK, conventional flat planting; FPS, straw mulch; FPP, transparent plastic film mulch; RFPS, ridge-furrow with plastic film-mulched ridge and straw-mulched furrow. Values are given as the means ± standard errors of means. Values followed by different letters in each column for each year represent significant differences (*P* < 0.05) between treatments based on LSD tests (n = 3).

### Economic benefit

As shown in Table 3, the mean annul input costs in the mulch treatments were higher than in CK, mainly due to labour. Because the same planting densities and fertilizer rates were used under all treatments, input costs for these were the same under each treatment. The same additional input costs were required in RFPS and FPP for plastic film ($123.7 ha^−1^), with no corresponding costs in FPS and CK. The mean annual labour input costs under RFPS, FPP and FPS were 23.39%, 13.88% and 7.10% higher than those under CK, respectively. Related expenses included ridge-furrow framework building and maintenance, plastic film management (soil preparation and mulching) and straw management (straw collection, chopping and mulching, etc.) in RFPS; plastic film management in FPP; and straw management in FPS.

**Table 3:** Economic benefit analyses of winter wheat production under various mulching cultivations over both growing seasons.

Years	Treatments	Input ($ ha^−1^)					Output ($ ha^−1^)	Output:input	Economic benefit ($ ha^−1^)
		Fertilizer	Seed	Film	Labor	Total			
2014–2015	CK	79.5	99.3	—	244.3	423.0	1,041.4	1.34:1	618.4
	FPS	79.5	99.3	—	261.6	440.3	1,125.5	1.39:1	685.1
	FPP	79.5	99.3	123.7	278.2	580.6	1,495.2	1.40:1	914.6
	RFPS	79.5	99.3	123.7	301.4	603.9	1,624.9	1.46:1	1,021.1
2015–2016	CK	79.5	99.3	—	244.3	423.0	995.8	1.28:1	572.8
	FPS	79.5	99.3	—	261.6	440.3	1,105.9	1.36:1	665.5
	FPP	79.5	99.3	123.7	278.2	580.6	1,452.9	1.36:1	872.3
	RFPS	79.5	99.3	123.7	301.4	603.9	1,568.8	1.41:1	964.9

CK, conventional flat planting; FPS, straw mulch; FPP, transparent plastic film mulch; RFPS, ridge-furrow with plastic film-mulched ridge and straw-mulched furrow.

Output refers to the revenue from the sale of grain. As indicated in Table 3, the average output was increased under the mulch treatments compared with CK. Specifically, RFPS was recorded the highest output over both years: $1,624.9 ha^−1^ in 2015 and $1,568.8 ha^−1^ in 2016. The output was lower for FPP ($1,495.2 ha^−1^ in 2015 and $1,492.9 ha^−1^ in 2016) and even lower for FPS ($1,125.5 ha^−1^ in 2015 and $1,105.9 ha^−1^ in 2016). In general, we found that higher input costs were associated with higher outputs, i.e., more economic benefit. The average annual economic benefit was greatest under RFPS, followed in order by FPP and FPS, relative to CK (66.72%, 50.01% and 13.38%, respectively).

## Discussion

Mulching cultivations are known as the critical approach to reduce soil evaporation and to improve soil water storage and crop water use efficiency, especially in dryland farming systems^36–38^. In our study, we showed that in the early growing stages, the SWS from 0–40 cm under the RFPS or FPP was significantly (*P* < 0.05) higher than under FPS or CK. However, in the late growing stages, the early gains became non-significant (*P* > 0.05) (Figs 1 and 2, Supplementary Fig. S1). These results are mainly due to the RFPS and FPP greatly decreasing evaporation in the early growing stages and strongly increasing the WUE in the later stages^14,39^. Furthermore, the increased shoot biomass with plastic film mulch greatly increased the WUE and hence reduced SWS during the season, and the decline in SWS occurred especially in the topsoil and subsoil layers^24,26,40^. FPS had a higher SWS than CK, although there were no significant differences (*P* > 0.05) between treatments in either of the two growing seasons. This result is consistent with previous studies^23,30,31^, indicating that the reduction in evaporation and increased water retention under straw mulch was limited and relatively short-lived because of the rapid degradation of the mulch with high microbial activity stimulated by good nutrient supply. From 41–60 cm, the SWS under RFPS was highest among all treatments over both growing seasons (*P* < 0.05). No significant differences (*P* > 0.05) were detected among FPP, FPS and CK (Fig. 1, Supplementary Fig. S1c and S1g). This is probably due to RFPS serving as a physical barrier, restricting runoff or evaporation of rainwater, which allow increased water percolation and storage in the soil^14,26^. From 61–100 cm, the SWS in mulch treatments were lower than in CK over both seasons, with significant differences (*P* < 0.05) between FPP and CK (Fig. 1, Supplementary Fig. S1d and S1h). Previous studies have shown that a high WUE might decrease the deep water percolation under mulching cultivations^24,41^; additionally, with heterogeneous and alternating moisture conditions, water deficit increases root growth in deeper layers (60–100 cm) or increases the numbers of lateral roots, which increase water uptake^42^. Excessive water consumption caused by root absorption and inter-row evaporation under plastic film mulch, resulting in the reduction of soil moisture content in deeper layers^43,44^, our study agrees with these results. Earlier studies have found that plastic film mulch are very effective in rain water harvesting and enabling better penetration of water to the depths accessible to the roots^18,45^. The rate of water consumption increased with increasing biomass to rates much higher than precipitation, so water consumption resulted in higher soil water depletion^14,44^. Our results showed that the amount of available SWS from 0–100 cm in RFPS or FPP was higher than in FPS and CK from 0 to 125 DAS; however, in RFPS and FPP, the SWS was strongly decreased between 125 and 250 DAS (Fig. 2).

The effects of mulches on soil temperature have been widely reported^13,27^. In general, the influence of a mulch on soil temperature depends on the balance between the ability of the mulch material to reflect and transmit solar energy^27^; therefore, soil temperature generally increases under a plastic film mulch and decreases under a straw mulch^30,31^. In our study, at the tillering (BBCH 21–28) and overwintering (BBCH 29–30) stages, soil temperature was considerably improved under RFPS and FPP compared with CK, with soil temperature being maximal under RFPS and slightly higher under FPS than under CK (Fig. 3b,c, Supplementary Table S1). These results agree with Chen *et al*.^14^ and Ramakrishna *et al*.^46^, who also found higher soil temperatures under plastic film mulch than under straw mulch. This may be due to small plant canopies allowing more sunlight to fall on the plastic film, quickly warming the topsoil; additionally, heat loss from the soil was lower under a mulch so soil temperature was better maintained at night^13^. Gao *et al*.^30^ have reported that a straw mulch decreased soil temperature in the very early season; this result is consistent with our study (Fig. 3a, Supplementary Table S1). While increased soil temperature and improved soil moisture conditions provide a good basis for early growth of winter wheat, soil temperature stress is common in the late season and can lead to unacceptable yield losses^27^. In our study, RFPS and FPP accelerated soil temperature rise at the stem elongation (BBCH 31–39) and milking (BBCH 73–77) stages, with the temperature increase under FPP exceeding that under RFPS and with the temperature under FPP being higher than under RFPS in the later season (Fig. 3c,d, Supplementary Table S1). These results agree with Bhardwaj *et al*.^27^ and Zhang *et al*.^13^ and may be attributed to the FPP receiving more solar energy with a bare inter-row increasing thermal conductance. Meanwhile, the straw in the furrow under RFPS had a higher albedo and lower thermal conductivity than bare soil, thereby reducing the amount of solar energy reaching the soil and the rate of temperature increase at the warmer stage^19,47^. It is for this reason that soil temperature in FPS was much lower than that in CK during the warm stage, resulting in negative effects on the early growth (Fig. 3a,d). In addition, the higher soil temperature under FPP suggests that soil water loss may have been higher than under RFPS, thereby reducing SWS and grain yield^34,48^.

In dryland farming systems, crop productivity depends to a great extent on rainfall, and the yield frequently correlates with rainfall at critical growth stages^49^. Mulching increases SWS and thus extends the period of water availability during dry spells^50^. Additionally, soil water loss through surface evaporation is reduced, and soil temperatures are more favourable; hence, both grain yield and WUE increases^14,30^. In our study, mulching cultivations increased grain yield and WUE, particularly under RFPS and FPP (Tables 1 and 2). This finding is consistent with those of Bhardwaj *et al*.^27^ and Lin *et al*.^37^, who attributed their results to surface cover and improvements in rainwater harvesting and soil moisture conservation, and the shading effect of the canopy under a mulch was greater than under CK. Our results demonstrated that among the various mulch treatments, RFPS gave the maximum yield and WUE, followed in the order by FPP and FPS, with all differences being significant (*P* < 0.05) (Tables 1 and 2). Earlier studies confirmed this result, also agreeing with the differences between the various mulching cultivations^30,43^. These results can be adequately explained by the better control of soil temperature in RFPS compared with that in FPP and FPS. Additionally, The RFPS decreased soil water loss by surface evaporation with its ridge-furrow, plastic film and straw mulch, with increases in WUE. As a result, the yield components were all increased, including: ears m^−2^, grain numbers ear^−1^ and grain weight (Table 1). In contrast with RFPS, under FPP was a bare inter-row and continuous high soil temperatures, and under FPS was a short-lived water evaporation and a rapid degradation of the mulch, increasing surface evaporation and decreasing transpiration and WUE (Fig. 2, Tables 1 and 2). These effects brought about corresponding reductions in the yield components and thus in the overall grain yields. Under RFPS, the straw increased soil organic matter and soil minerals (i.e., available N, P and K), which led to the yield and WUE increases^14,34^.

Mulching and conservation tillage contribute to raise economic benefits in dryland farming systems^51^. Moreover, as shown by a simulation study, long-term mulch and conservation tillage are potentially sustainable methods for enhancing wheat production and improving water balance^52^. To assess the economic effectiveness under the various mulch treatments, financial input and output were calculated during two growing seasons^9,40^. Our study evaluated the economic returns of wheat grain yield based on a five-year average of local prices. The results demonstrated that the mean annual economic benefit was greatly increased in mulching cultivations compared with that in CK. Under RPFS, the economic benefit was increased by 66.72%; under FPP, it was increased by 50.01%; and under FPS, it was increased by 13.38%. Additionally, relative to the other treatments, RFPS showed the highest mean output:input ratio (1.44:1) over both seasons. The increased economic benefits of RFPS are satisfactorily explained by the higher values of SWS and WUE and the more moderate soil temperatures (Figs. 1 and 2, Tables 2 and 3).

## Conclusion

Dryland winter wheat production is obviously influenced by rainfall and by extreme climatic events (droughts, extreme temperatures and other extreme conditions); thus, it is necessary to provide an inexpensive and effective approach to address adverse environmental conditions in dryland farming systems. Mulching cultivation is a promising management tool for improving crop production. In our study, during two growing seasons of winter wheat, RFPS, FPP and FPS improved the SWS, WUE and grain yields of winter wheat, respectively, in the order of RFPS > FPP > FPS. In cold seasons, compared with CK, the soil temperatures of RFPS and FPP were increased, but that of FPS was decreased. We concluded that RFPS was the most effective management (better than FPP and FPS), as it best controlled soil moisture and soil temperature and resulted in increased grain yield with higher WUE and increased economic benefits. In the long term, ridge-furrow planting with plastic film-mulched ridge and straw-mulched furrow has considerable potential to improve winter wheat productivity, reduce impacts of extreme weather and hence increase food security and the economic prospects of farmers in dryland farming systems.

## Materials and Methods

### Experimental site

**Figure 4:**
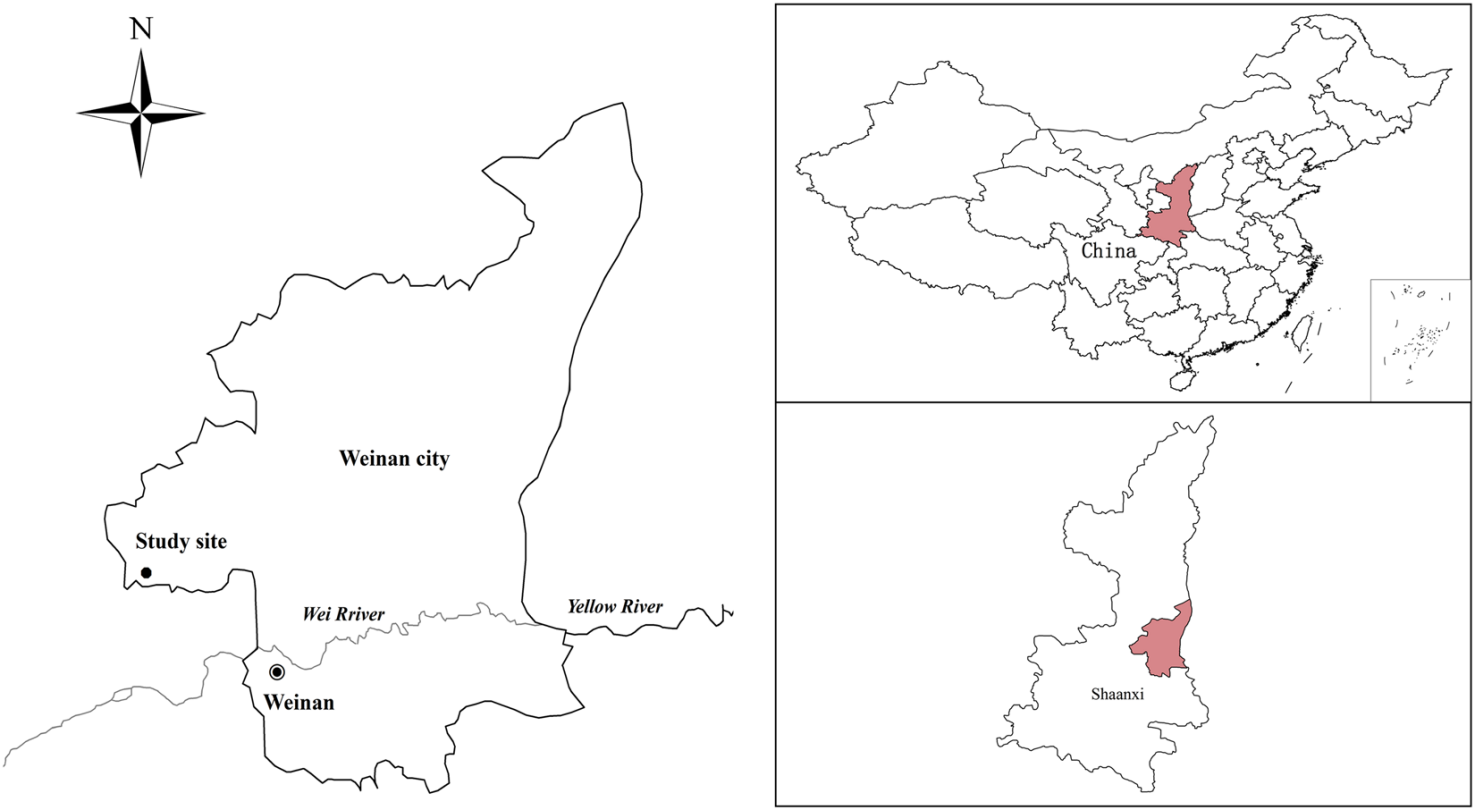
Location of the study site in Weinan City, Shaanxi Province, China. The maps of Weinan, Shaanxi and China from Google earth (https://ditu.google.cn) were captured and used for drawing vector graphics with an ArcGIS Desktop Basic Version (https://store.esri.com/content/esri/en-us/arcGISDesktop.html).

**Figure 5:**
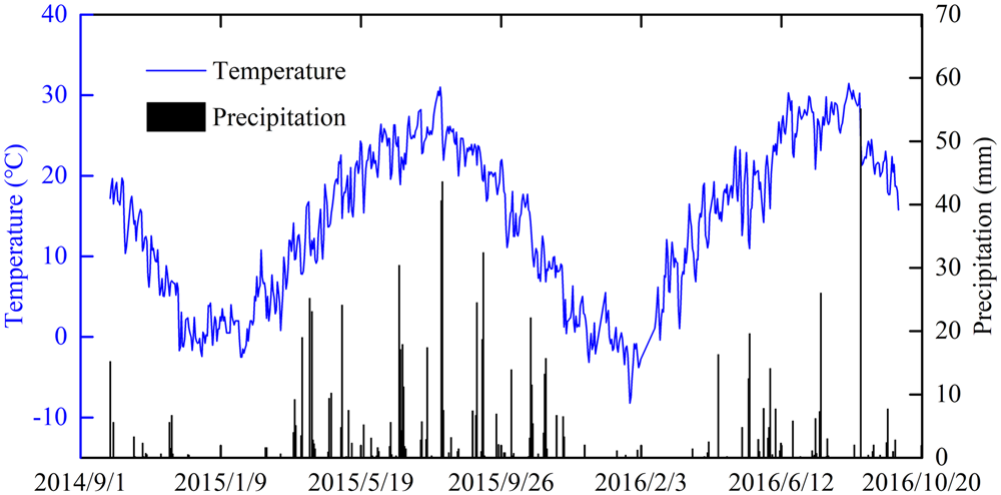
Air temperature and precipitation during the experimental period.

A two-year field experiment with a winter wheat-fallow planting system was conducted from September 2014 to June 2016. The experiment was conducted at the Fuping Comprehensive Experiment Station of Northwest A&F University (34°74′N and 109°10′E) in Fuping Country, Weinan City, Shaanxi Province, China (Fig. 4). The region is located in the southern Loess Plateau, with an elevation of 472 m and a mean annual precipitation of approximately 550 mm, with 70% of the rainfall in July, August, and September; water scarcity is a serious problem in this region^16,53^. The mean annual air temperature is 12.5 °C, and the mean annual evaporation is 1826.7 mm. The soils at the experiment site are clay loams and classified as Typic Calciustepts (Inceptisols) according to the USDA soil taxonomy system, belonging to typical soil type^54^. The basic soil properties and water characteristics at the beginning of the study are shown in Table 4. Meteorological data collected during the two-year experimental period are shown in Fig. 5.

**Table 4:** The basic soil properties and water characteristics at the experimental site (2013).

SD (cm)	SOM (g kg^−1^)	TN (g kg^−1^)	NO_3_^–^-N (mg kg^−1^)	NH_4_^+^-N (mg kg^−1^)	AP (mg kg^−1^)	pH	BD (g cm^−3^)	DUL (cm^3^ cm^−3^)	WLL (cm^3^ cm^−3^)
0–20	11.42	0.58	9.18	2.31	9.35	8.34	1.32	0.27	0.08
21–40	8.25	0.51	6.30	1.82	5.50	8.39	1.37	0.27	0.07
41–60	8.34	0.43	1.56	0.67	4.08	8.46	1.42	0.26	0.08
60–80	7.44	0.42	0.88	0.56	1.96	8.52	1.41	0.27	0.08
80–100	5.53	0.34	0.81	0.62	1.84	8.55	1.44	0.26	0.08

SD, soil depth; SOM, soil organic matter; TN, total nitrogen; AP, available phosphorus; BD, bulk density; DUL, drainage upper limit; WLL, water lower limit.

### Experimental design and field management

**Figure 6:**
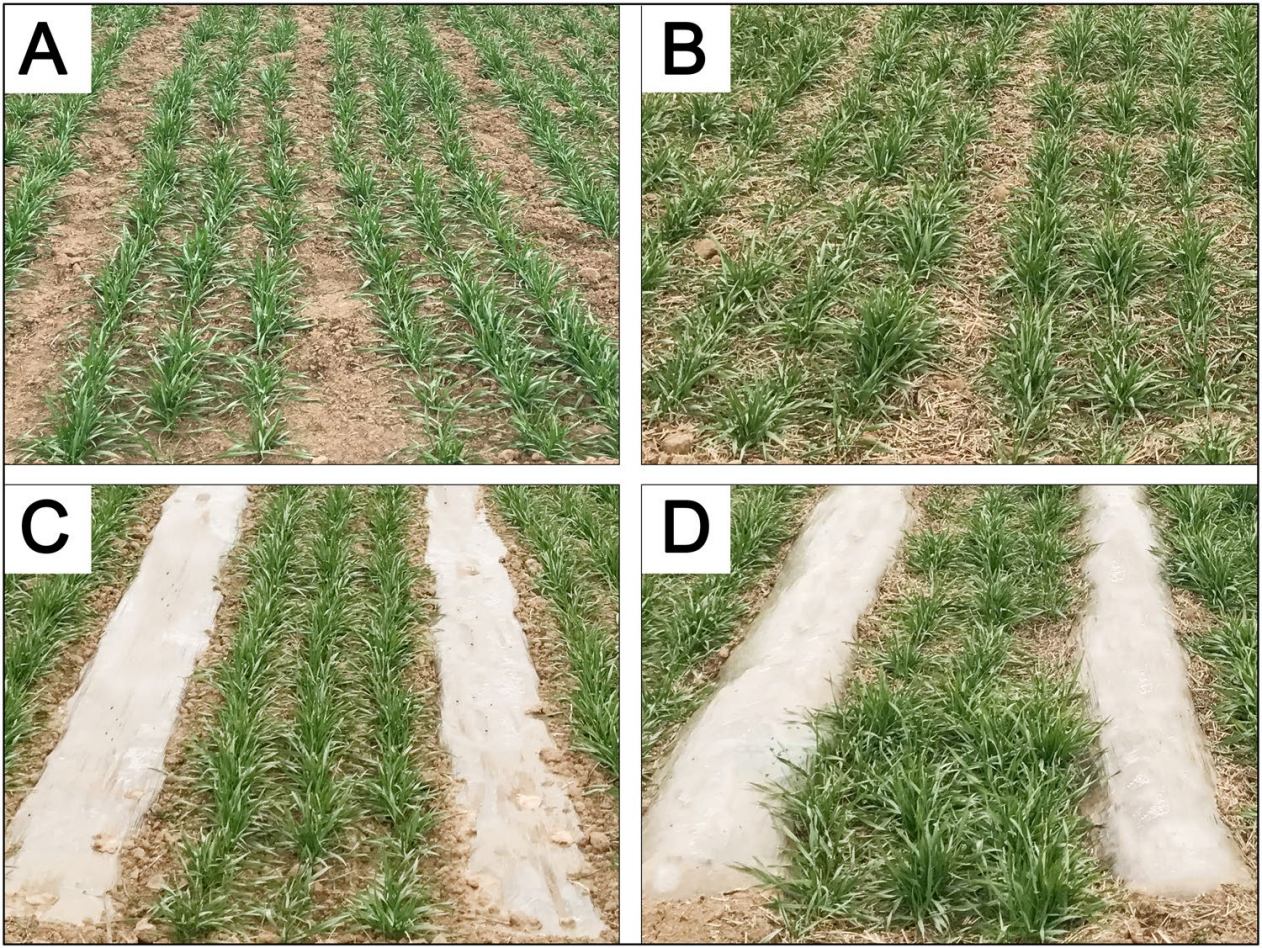
Plot layouts of different field management regimes in this study. (**A**), conventional flat planting (CK); (**B**), straw mulch (FPS); (**C**), transparent plastic film mulch (FPP); (**D**), ridge-furrow with plastic film-mulched ridge and straw-mulched furrow (RFPS).

Four treatments were imposed: (1) conventional flat planting (CK), (2) straw mulch (FPS), (3) transparent plastic film mulch (FPP) and (4) ridge-furrow with plastic film-mulched ridge and straw-mulched furrow (RFPS) (Fig. 6). Treatments were replicated three times and distributed across 12 plots each of size 18 m × 5 m = 90 m^2^, with completely randomised design. Fifteen days before sowing, the plastic films (0.008 mm thick, 45 cm wide) were applied in the FPP plots, with a distance between two rows of plastic films of 60 cm. Meanwhile, the ridges (35 cm wide and 15 cm high) were built and covered by plastic films (0.008 mm thick and 45 cm wide) in the RFPS plots, and the size of furrows was 60 cm wide. No additional field managements were applied in the CK and FPS plots. Fertilizers (135 kg N ha^−1^, 90 kg P_2_O_5_ ha^−1^) were broadcast in the CK and FPS plots, between two lines of plastic films in the FPP plots and in the furrows in the RFPS plots, which incorporated to a depth of approximately 15 cm by ploughing. Winter wheat was directly sown in the CK and FPS plots and sown between two lines of plastic films in the FPP plots and in the furrows in the RFPS plots. After sowing, air-dried wheat straws (approximately 15% moisture content, moisture determination using oven-drying method^55^) from the previous season were chopped into approximately 5–10 cm lengths and were spread evenly over the plots in the FPS and in the furrows of the RFPS.

In this study, the sowing dates of winter wheat were October 3, 2014, and October 5, 2015. The planting density was 180 kg ha^−1^. The harvest dates were June 5, 2015, and June 9, 2016. No irrigation was applied to any treatments during the two-year experimental period. Field management measures included weeding and insecticide spraying, in line with local practice.

### Sampling, measurements and calculations

Mean daily air temperature and rainfall were recorded by an automatic weather station (ZQZ-A Automatic Weather Station, Jiangsu, China) approximately 100 m from the experimental plots, which could record the changes in the hourly air temperature and rainfall. The meteorological data were collected by the ZQZ-A Data Acquisition System.

Before our study (in September 2013) in this experimental plot, soil samples from four points were collected by a 50-mm-diameter steel core sampling tube at 20 cm depth intervals down to 100 cm. The soil samples at the same depths were mixed to produce a composite sample and were used for analysing the basic chemical properties (soil organic matter, nutrients and pH). The chemical properties were determined according to Unger^56^ and Klute^57^. The soil bulk density (BD) and water characteristics (drainage upper limit and water lower limit) in the 0–100 cm depth (at 20 cm depth intervals) were determined using the core method^58,59^, where the volume of the stainless ring employed was 200 cm^3^. Three core samples were collected randomly in experimental plot, the averages were used as the basic BD, drainage upper limit (DUL) and water lower limit (WLL).

During the winter wheat growing season in 2014–2015 and 2015–2016, three soil cores for each plot replicate were collected by a 50-mm-diameter steel core sampling tube every 25 days, at 20 cm depth intervals down to 100 cm. The soil cores were categorized (0–20, 21–40, 41–60, 61–80 and 81–100 cm) according to depth and were immediately placed into aluminum boxes for determining gravimetric soil water (GSW). Additionally, the soil samples at critical growth stages, i.e., the seedling (BBCH Growth Stage 17), stem elongation (BBCH Growth Stage 33) and milking (BBCH Growth Stage 73) stages (the phenological growth stages and BBCH-identification keys were determined based on Witzenberger *et al*.^60^), were used for comparing the differences in soil moisture between treatments. GSW was measured according to Siahpoosh *et al*.^61^. For soil BD, soil samples were collected with three points for each plot replicate in 0–100 cm depth (at 20 cm depth intervals) after wheat harvest in 2015 and 2016, and the methods of sample collection, determination and calculation were according to Mccarty *et al*.^57^. SWS was calculated according to equation (1)^40^, total water consumption (ET, evapotranspiration) was calculated using equation (2)^62^, and WUE was estimated according to equation (3)^16^.

1 $$SWS(mm)=GSW\times BD\times SD$$

where ${GSW}$ is soil gravimetric moisture content (%), ${BD}$ is bulk density (g cm^−3^), and ${SD}$ is soil depth (mm).

2 $$ET(mm)={P}_{e}+{\Delta }SWS$$

where ${P}_{e}$ is effective rainfall (mm), and $\Delta {SWS}$ is soil water depletion from the root zone during the growing season.

3 $$WUE(kgh{a}^{-1}m{m}^{-1})=Y/ET$$

where $Y$ is grain yield (kg ha^−1^).

A Time Domain Reflectometry (CR1000, Campbell Scientific, USA) system with four probes was installed for monitoring and reporting the soil temperature. Four probes were installed in the middle of one of three replications at a 10 cm depth and could monitor mean topsoil temperature every 24 h. Soil temperature data were collected by a wireless data transmission module (SHUNCOM, Shanghai, China). In this experiment, the sowing-seedling (BBCH Growth Stage 00–19), tillering (BBCH Growth Stage 21–28), overwintering (BBCH Growth Stage 29–30), stem elongation (BBCH Growth stage 31–39) and milking (BBCH Growth Stage 73–77) stages were used for estimating the dynamic changes of mean daily soil temperature in all treatments (the phenological growth stages and BBCH-identification keys were determined based on Witzenberger *et al*.^60^).

Wheat grain yield samples from the middle three rows (equal to 4.14 m^2^) of each plot were collected by hand on June 5, 2015, and June 9, 2016, respectively. The number of wheat ears m^−2^ was determined by the total yield samples of each plot divided by 4.14 m^2^. The wheat grain yield samples of each plot were threshed by hand, and grains were air-dried (approximately 12% moisture content) to determine grain yields, in which the grain weight (the thousand-grain weight divided by 1000) was also determined. Meanwhile, additional fifteen wheat ears from each plot (non-edge areas) were randomly sampled to determine the grain numbers ear^−1^. The grain numbers m^−2^ were calculated by multiplying the numbers of ears m^−2^ by grain numbers ear^−1^.

Economic benefit was analysed by the difference and ratio between input and output^9,13,40^. In this experiment, inputs were divided into two main parts, including investments in labour and finance. The labour involved was in land preparation, ridge-furrow building, laying plastic film and straw mulches, sowing, field management (pesticide spraying, weeding, plastic film maintenance and removal, etc.) and harvesting. In addition to the experimental input costs were the costs of fertilizer, plastic film and seed. The output was assessed on the basis of the grain yield multiplied by the 5-year average of market price (0.27 $ kg^−1^) of local winter wheat grain.

### Statistical analyses

All data were collected together and sorted using Excel 2013. Data were analysed by one-way ANOVA using SPSS version 19.0 for Windows. The significance of the differences between treatments was compared using the Fisher’s LSD method at *P* = 0.05 level.

## Electronic supplementary material

Supplementary Information
