# Supplementary material for: Ridge-furrow with plastic film and straw mulch increases water availability and wheat production on the Loess Plateau
Source: Sci Rep. 2018 Apr 25;8:6503. doi: 10.1038/s41598-018-24864-4 (PMC5917030; doi:10.1038/s41598-018-24864-4)
Supplement: Supplementary file 1 — Supplementary Information [file 41598_2018_24864_MOESM1_ESM.pdf]

**Ridge-furrow with plastic film and straw mulch increases water availability and wheat production on the Loess Plateau**

Gaoyuan Liu[1,2], Yuhuan Zuo[1], Qi Zhang[1], Lili Yang[1], Erlong Zhao[1,2], Lianyou Liang[1,2], Yan' an Tong[1,2,*]

[1] College of Natural Resources and Environment, Northwest A&F University, Yangling 712100, Shaanxi, China.

[2] Fuping Comprehensive Experiment Station of Northwest A&F University, Weinan 714000, Shaanxi, China.

[*]Corresponding author. E-mail address: tongyanan@hotmail.com.

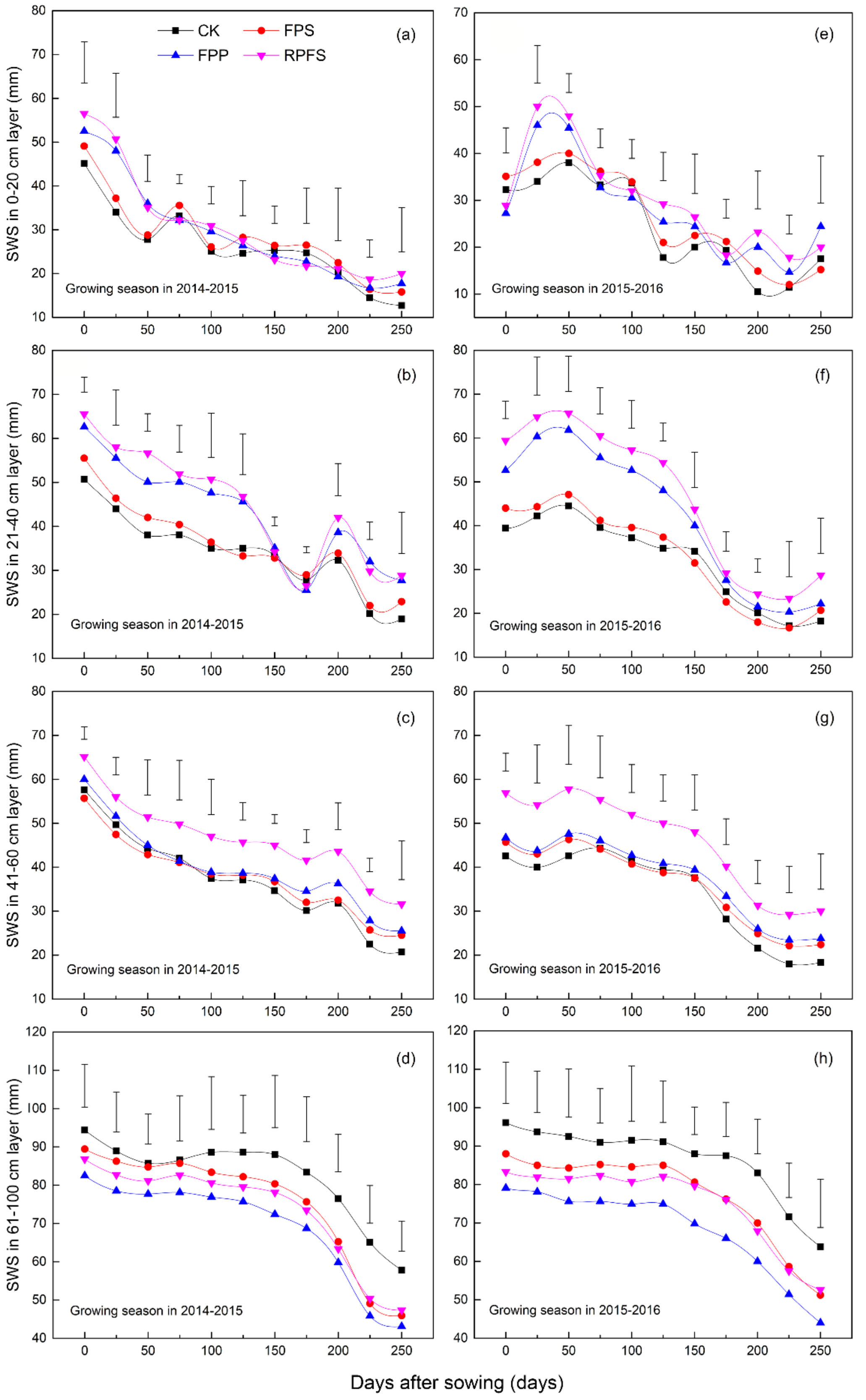


**Supplementary Figure S1. Soil moisture dynamics from various soil depths under different mulching cultivations over two growing seasons.** (a), (b), (c) and (d) represent soil water storage from different soil layers in 2014-2015, and (e), (f), (g) and (h) represent soil water storage from different soil layers in 2015-2016, respectively. CK, conventional flat planting; FPS, straw mulch; FPP, transparent plastic film mulch; RFPS, ridge-furrow with plastic film-mulched ridge and straw-mulched furrow. Vertical bars represent $LSD_{0.05}$ between different treatments (n = 3).

| Years | Treatments | Sowing-seedling | Tillering | Overwintering | Stem elongation | Milking |
|---|---|---|---|---|---|---|
| 2014-2015 | CK | 16.9 (1.4) | 6.3 (2.6) | 1.9 (1.3) | 11.1 (1.1) | 16.2 (1.3) |
| | NPS | 16.3 (1.2) | 7.0 (2.4) | 2.2 (1.3) | 10.7 (1.1) | 15.5 (1.1) |
| | NPP | 18.0 (1.2) | 7.7 (2.3) | 3.2 (1.4) | 12.3 (1.3) | 17.7 (1.3) |
| | RFPS | 18.1 (1.1) | 9.1 (2.2) | 4.4 (1.2) | 12.6 (0.9) | 17.2 (1.1) |
| 2015-2016 | CK | 16.9 (1.0) | 6.3 (2.0) | 0.2 (1.1) | 11.0 (1.7) | 16.9 (1.4) |
| | NPS | 16.4 (0.9) | 6.7 (1.8) | 0.4 (1.0) | 10.5 (1.7) | 16.3 (1.4) |
| | NPP | 18.0 (1.0) | 7.2 (1.9) | 1.1 (0.8) | 11.8 (1.7) | 18.5 (1.4) |
| | RFPS | 18.6 (0.7) | 8.2 (1.6) | 2.2 (0.9) | 12.4 (1.4) | 18.1 (1.2) |

**Supplementary Table S1. The variation of daily mean soil temperatures at 10 cm soil depth under different mulching cultivations over two growing seasons.** CK, conventional flat planting; FPS, straw mulch; FPP, transparent plastic film mulch; RFPS, ridge-furrow with plastic film-mulched ridge and straw-mulched furrow. Values are given as the means (standard errors) during different growing stages.
